# Controlling the growth of nanosized titania *via* polymer gelation for photocatalytic applications†

**DOI:** 10.1039/d0ra03312j

**Published:** 2020-05-21

**Authors:** Yousra El Jemli, Mohammed Mansori, Oscar Gonzalez Diaz, Abdellatif Barakat, Abderrahim Solhy, Karima Abdelouahdi

**Affiliations:** IMED-Lab, FST, Cadi Ayyad University Marrakech Morocco abdelouahdi@gmail.com; Grupo de Fotocatálisis y Espectroscopía para Aplicaciones Medioambientales (Grupo FEAM), Unidad Asociada al CSIC, Dpto Química, Instituto de Estudios Ambientales y Recursos Naturales i-UNAT, Universidad de Las Palmas de Gran Canaria Campus Universitario de Tafira 35017 Las Palmas Spain; IATE, Montpellier University, INRAE, Agro Institute 34060 Montpellier France; Mohammed VI Polytechnic University Ben Guerir Morocco

## Abstract

Nanocrystalline titania was synthesized by a simple, innovative and eco-friendly gelation method by using biopolymers (polysaccharides). The effect of the gelling agent, such as carboxymethylcellulose (CMC) or alginate (Alg), and the drying routes (conventional drying at room temperature, or freeze-drying) on the properties and photocatalytic performances of nanostructured TiO_2_ was examined. The crystallographic structures, and textural and morphological characteristics were investigated by thermogravimetric analysis (TGA), X-ray diffraction (XRD), Fourier transform infrared spectroscopy (FTIR), scanning electron microscopy with energy dispersive spectrometry (ESEM-FEG-EDS), transmission electron microscopy (TEM), UV-vis diffuse reflectance spectroscopy (DRS) and N_2_ adsorption/desorption isotherms. The as-synthesized samples were fully crystallized and appeared to be highly phase-pure anatase or mixed titania polymorphs, and have a quasi-spherical shape with a particle size ranging from 10.34 to 18.07 nm. Phase-pure anatase was obtained by using alginate as the gelling agent, whereas CMC's gelation promotes mixed structures. The presence of rutile phase results in a lower bandgap value of 3.04 eV corresponding to 408 nm. Thus, the material absorption wavelength shifts slightly from the UV (190–380 nm) to visible region (380–750 nm). The drying process also affects TiO_2_ properties. The lyophilization route improves the oxide's specific surface area, and also its photocatalytic properties verified during Orange G dye photodegradation study.

## Abstract 2

Nanocrystalline titania was synthesized by a simple, innovative and eco-friendly gelation method by using biopolymers (polysaccharides).

## Graphical abstract

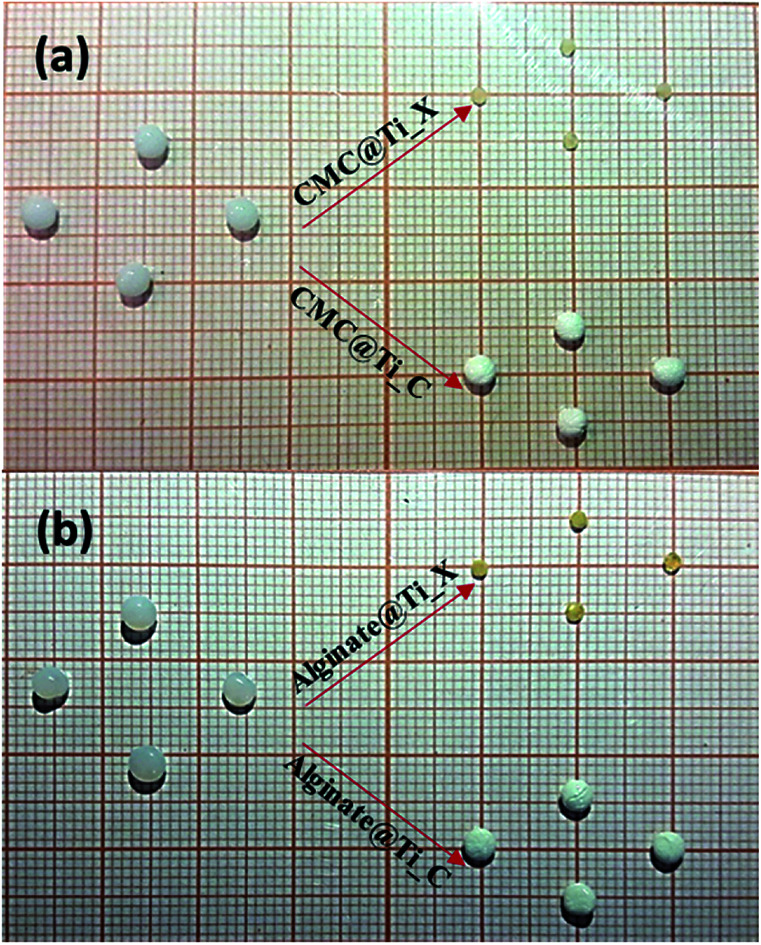

## 1. Introduction

Titania (TiO_2_) stands out among the widely studied semiconductor materials.^1–3^ In addition to its excellent physicochemical properties, chemical stability in a broad pH range and low cost, this oxide is environmentally benign.^2–11^ TiO_2_ has many high-tech applications such as photocatalysis, batteries, super capacitors, sensors and drug formulations.^5,12–18^ TiO_2_ occurs in four commonly known crystal forms: anatase, rutile, brookite and bronze.^19–22^ Anatase and rutile both have tetragonal structures, whereas the brookite crystal system is orthorhombic, and bronze is monoclinic.^20,21,23^ Anatase has a bandgap of 3.2 eV (385 nm), while the rutile phase has a smaller bandgap of 3.0 eV (410 nm).^3^ From a photocatalytic application point of view, anatase is the better candidate in comparison to rutile.^23,24^ It must however be noted that the presence of a mixture anatase–rutile or anatase–brookite phases in a well-defined ratio enhances photocatalytic performances.^23–27^ This can be explained by supposing to extend the photoactive response to the visible region, harvest more light, and stabilize the charge separation by electron transfer from rutile to anatase trapping sites slowing down the recombination.^26,28^

The physicochemical properties of TiO_2_, mainly its crystalline structure, bandgap energy and photocatalytic properties depend strongly on its synthesis method.^29^ The most common approaches can be classified into physical, wet-chemical, and biological synthesis processes.^30^ The wet-chemical route was already reported in order to control of oxide's particle size, shape, and stoichiometry.^21,31,32^ The sol–gel method ranks among the most investigated synthesis route.^33,34^ Biswas and co-workers prepared a pure anatase phase with a cubic shape *via* sol–gel method for electrochemical sensor applications.^34^ Mutuma *et al.* adopted the same method in order to evaluate the photocatalytic activity of mesoporous anatase–brookite and anatase–brookite–rutile TiO_2_ nanoparticles.^33^ In addition, we must also mention hydro-solvothermal route, which is another important method for titania design. Thus, Cano-Casanova *et al.* prepared TiO_2_ particles into spherical morphology under mixed-phases of anatase–rutile and anatase–brookite.^35^ Mamaghani *et al.* designed porous and interconnected architectures of anatase phase *via* the solvothermal route.^36^ Recently, considerable efforts were achieved in order to develop sustainable and eco-friendly procedures for oxides design.^37–39^ Surya *et al.*, prepared TiO_2_ by using *Jatropha curcas* leaf extracts for the photocatalytic degradation of tannery wastewater.^40^ Moreover, TiO_2_ was synthesized from *Cymbopogon proximus* in order to use it for Rhodamine B photodegradation and the study suggested its possible use for drinking water purification.^41^

On the other hand, TiO_2_ performances closely related also on the drying process used, which can directly impacted to oxide's porosity.^42^ The possibility of preserving porosity and avoiding capillarity collapse, improves, amongst others, the material specific surface area, and then in some cases enhances its photoactivity.^42–44^ To this end, exploring novel innovative technologies for well-controlled growth of titania, especially: its shape, its size, its morphology, its structure, and its specific surface area, remains a challenge.^21,45,46^ Eco-design and bio-inspired methodologies for the nanostructured TiO_2_ preparation have of great importance due to the growing need for contemporary society to develop sustainable approaches.^46^

In this study, we report for the first time the effect of the biopolymer (CMC *vs.* alginate) and the drying method on the crystalline phase (anatase, rutile, brookite), morphology, optical and photocatalytic properties of TiO_2_ nanoparticles prepared *via* the gelation of those biopolymers. We successfully elaborated nanocrystalline titania, *via* gelation of CMC and alginate by Ti^4+^ cations. CMC and alginate act as a template for crystal nucleation and growth, facilitates the formation of porous nanostructured oxides with well-controlled particle size and shape.^39,47^ The hybrid materials (xerogels and cryogels) were characterized by TGA to select the annealing temperature in order to well-crystallize titania. Alginate provided a pure anatase phase, whereas samples obtained by gelling CMC allows mixed-phase: anatase–rutile and anatase–brookite. The presence of a small amount of rutile phase resulted in a lower band gap and shifted the material absorption towards the visible region. Thus, as-prepared samples were tested for the photodegradation of Orange G. Mixed phases showed the highest activities, which is further improved by oxides obtained *via* the freeze-drying of hydrogel-beads.

## 2. Experimental section

### 2.1 Materials

Nanocrystalline titania was synthesized by using TiCl_4_ (99%, Merck KGaA) as precursor of titanium. CMC with a molecular weight of 90 000 g mol^−1^ and alginate (Sigma Aldrich) were used as gelling agents. The anionic Orange G (Merck KGaA) was used as model pollutant for decolorization of aqueous solutions containing this azo-dye (photocatalytic study). Ethanol (absolute grade, 99.9%), and ultra-pure water were used for hydrogel-beads elaboration.

### 2.2 Preparation of porous nanostructured titania

In a typical procedure, titanium tetrachloride (99% TiCl_4_), used as the starting material, was first stabilized in absolute ethanol and then subjected for the preparation of titanium(iv) precursor solution at 0.1 M. Biopolymer solution (5 wt%) was prepared by dissolving 5 g of CMC or alginate in 100 mL of ultra-pure water under vigorous stirring at room temperature, forming an homogeneous viscous solution. The mixture was stirred for, at least, 10 h to ensure complete dissolution. Then, the biopolymer gel (5%: w/w) was added dropwise, at room temperature, into Ti^4+^ precursor solution *via* a syringe with a 0.8 mm diameter needle and constantly stirred for 2 h. The obtained hydrogel beads (biopolymer@Ti) were washed three times consecutively with ultra-pure water. Then, the beads were dried, using two different procedures, either at room temperature forming xerogel beads or freeze dried forming cryogel beads. Thereafter, a heat treatment of xerogel and cryogel beads was performed under air to remove organic matrix and to obtain pure titania. In both cases, the obtained beads were annealed at 500 °C for 4 h. In the present study, we investigated the influence of the gelling agent and the drying manner on the properties of titania. The different elaborated hybrid and oxide materials were denoted as shown in Table 1.

**Table 1:** References of as-prepared samples according to their different synthesis and post-synthesis conditions^a^

Gelling agent	Conventional drying		Freeze drying	
	Before calcination	After calcination	Before calcination	After calcination
CMC	CMC@Ti_X	TiO_2__X_cmc_	CMC@Ti_C	TiO_2__C_cmc_
Alginate	Alginate@Ti_X	TiO_2__X_alg_	Alginate@Ti_C	TiO_2__C_alg_

a X: xerogel; C: cryogel; cmc: carboxymethylcellulose; Alg: alginate.

### 2.3 Characterization techniques

The hydrogel beads were lyophilized under vacuum (0.01 bar, −60 °C) using Martin Christ Alpha 1-2 LD Plus freeze dryer. FT-IR spectra of the prepared samples were measured using KBr in the range of 4000–400 cm^−1^ on a Bruker VERTEX 70. TGA was conducted under air in a Labsys Evo apparatus with a 10 °C min^−1^ ramp between 25 and 1000 °C. XRD patterns were obtained at room temperature on a Rigaku SmartLab X-ray diffractometer using Cu-Kα radiation in Bragg–Brentano geometry (*θ*–2*θ*). The morphologies of the prepared samples were observed by environmental scanning electron microscope equipped with a Schottky field emission gun and an energy-dispersive spectrometer (ESEM-FEG-EDS, Quattro S, Thermo Fisher). TEM micrographs were obtained on a Tecnai G2 microscope at 120 kV. UV-DRS spectra was carried out on a Varian Cary 5 spectrometer equipped with an integrating sphere using polytetrafluoroethylene as a reference in order to study the optoelectronic properties of the elaborated oxides and was recorded at room temperature in the wavelength ranging from 300 to 700 nm. The surface charge of the oxides was measured on a Zetasizer (Nano ZS, Malvern Instruments Ltd, 7.12). The gas physisorption isotherms data were collected using a Micromeritics FlowSorb III Surface Characterization Analyzer using N_2_. Prior to N_2_ sorption, all samples were degassed at 300 °C overnight. The specific surface areas were determined from the nitrogen adsorption/desorption isotherms (at −196 °C), using the BET (Brunauer–Emmett–Teller) method. Dye solution concentration was measured using a UV-vis spectrophotometer (UV-2600, Shimadzu).

### 2.4 Photocatalytic dye-degradation experiments

The photocatalytic activity of as-prepared TiO_2_ was evaluated based on the photo-decomposition rates of aqueous Orange G (OG, a typical mono-azoic dye)^48^ at room temperature. The reaction was performed in a batch quartz reactor (40 × 20 × 36 mm^3^) and was illuminated by UV light using a lamp (Philips HPL-N 125W) emitting at 365 nm. The distance to the lamp was adjusted to irradiate the reactor with a photon flux of 1.05 mW cm^−2^, which is the intensity of UVA of the standard terrestrial solar spectral.^49^ Direct photolysis for more than 24 h of a solution in the absence of the photocatalyst was found to be negligible (white test). In a typical process, 25 mg of the photocatalyst was suspended in an OG aqueous solution (10^−5^ M, 25 mL). Prior to UV irradiation, each suspension was magnetically agitated with an inert Teflon magnetic stirrer in darkness for 1 h to reach the adsorption–desorption equilibrium. During the degradation experiments, a certain volume of the solution was taken at selected time intervals and filtered through a 0.45 μm Syringe Filters (Millex® HA). The concentration of the filtrate (OG solution) was then determined by measuring the absorbance at 478 nm *via* the UV-vis spectrophotometer.

## 3. Results and discussion

### 3.1 Elaboration and characterization of hybrid-beads

#### 3.1.1 Preparation of hybrid-beads

**Fig. 1:**
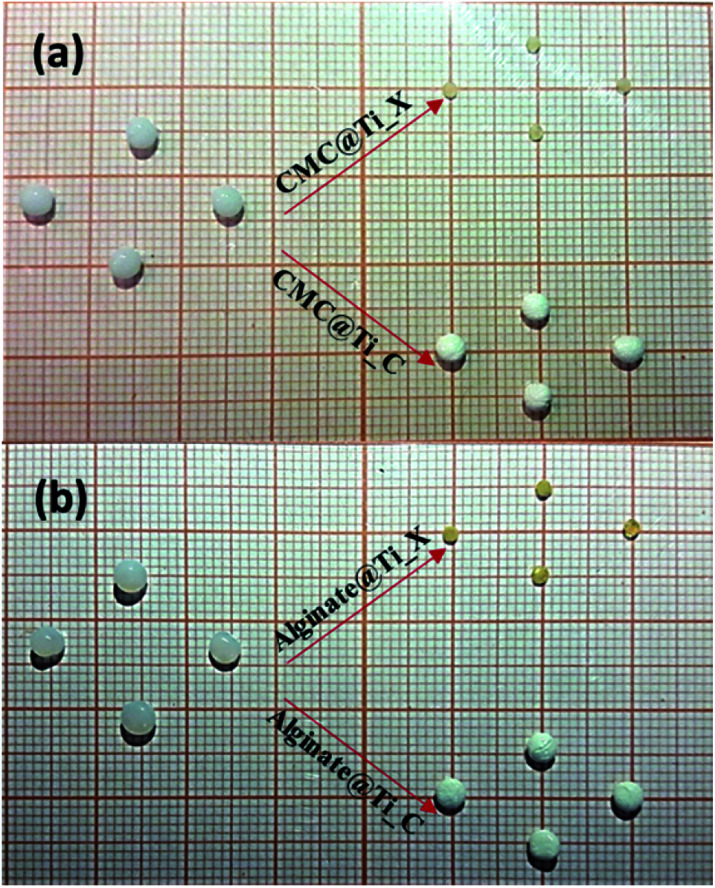
Digital images of hydrogel, xerogel-, and cryogel-beads synthesized *via* gelation of biopolymers: (a) CMC and (b) alginate.

The Fig. 1 presents the digital images of hybrid-beads, before (hydrogels) and after drying process (xerogels or cryogels), prepared by using CMC: CMC@Ti_X, CMC@Ti_C (Fig. 1a) and alginate: alginate@Ti_X, alginate@Ti_C (Fig. 1b). As it is shown in the Fig. 1, the conventional drying manner induces the contraction of the hydrogel spheres, which shrink up to 60–70% of volume, while the freeze-drying process preserves the form, the volume and the texture of hydrogel beads and thus maintains the spatial dispersal of biopolymers fibrils reticulated by Ti^4+^ in the hybrid-beads. It is evident that during the conventional evaporation process, a capillary tension is developed due to the liquid–gas–solid interface, which causes the capillarity's collapse of the hydrogel network. However, the lyophilization removes frozen-water *via* sublimation under vacuum. This can be achieved without the deformation of three-dimensional structure and also porous structure of cross-linked biopolymer, thus avoiding the capillarity's collapse and minimizing the cracking, and other processes related to drying mode.

#### 3.1.2 Microstructural analysis of hybrid-beads by FTIR

**Fig. 2:**
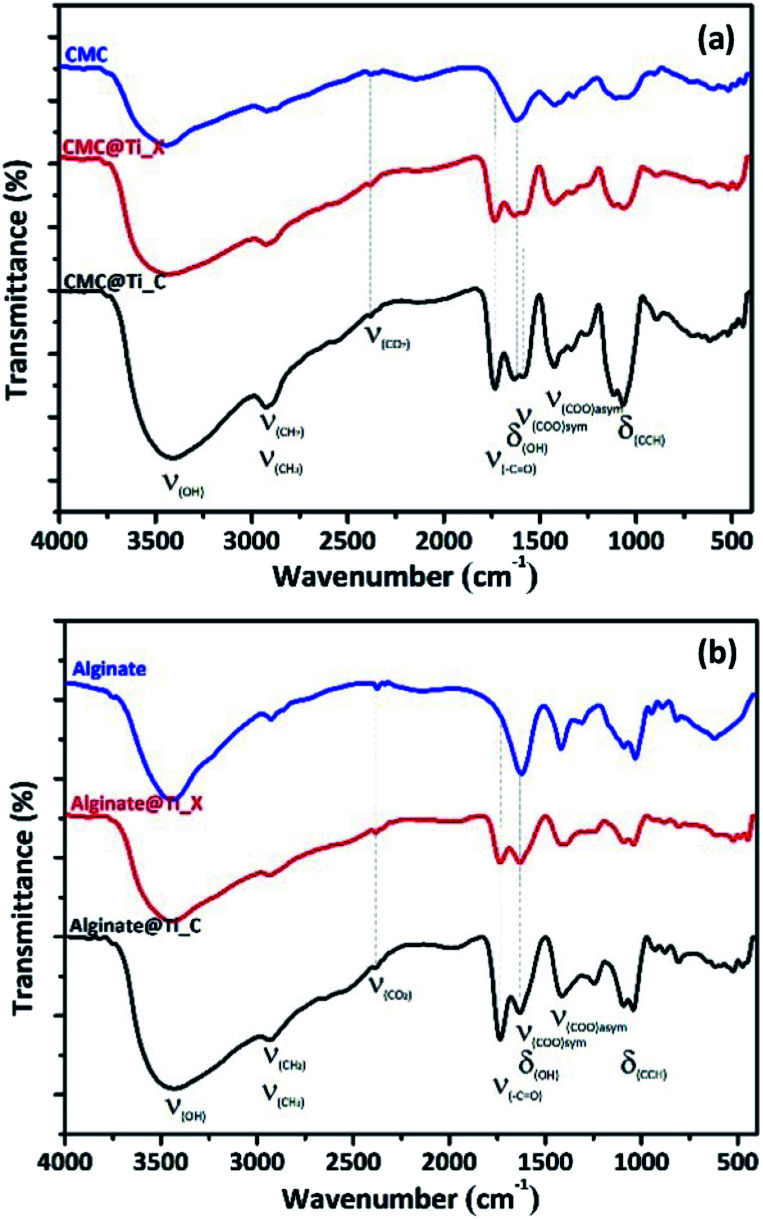
FTIR spectra of hybrid-beads prepared by using (a) CMC and (b) alginate.

Fig. 2 shows the FT-IR spectra of biopolymers (CMC and alginate), and the as-prepared hybrid-beads (xerogels and cryogels) according to drying process. The IR spectrum of both biopolymers can be assigned by analogy to the literature.^50,51^ FTIR analysis of the hybrid-beads (non-calcined samples) indicated the appearance of the characteristic peaks of the biopolymers used with slight modifications (enhanced intensities) related to cross-linking biopolymer. It must be emphasized that an additional peak at 1733 cm^−1^ was observed in all hybrid materials, attributed to carbonyl stretching (–C=O group). Also, the characteristic peak at 1104 cm^−1^ shifted slightly towards lower frequencies, due to reticulation of biopolymers fibrils by metal cations (Ti^4+^) in the hybrid-beads. As it was observed from FTIR spectra, there are no additional characteristic vibrational bands ascribed to the crystalline phase of titania, which suggests that all xerogel and cryogel samples are still amorphous.

#### 3.1.3 Morphological analysis of hybrid-beads

**Fig. 3:**
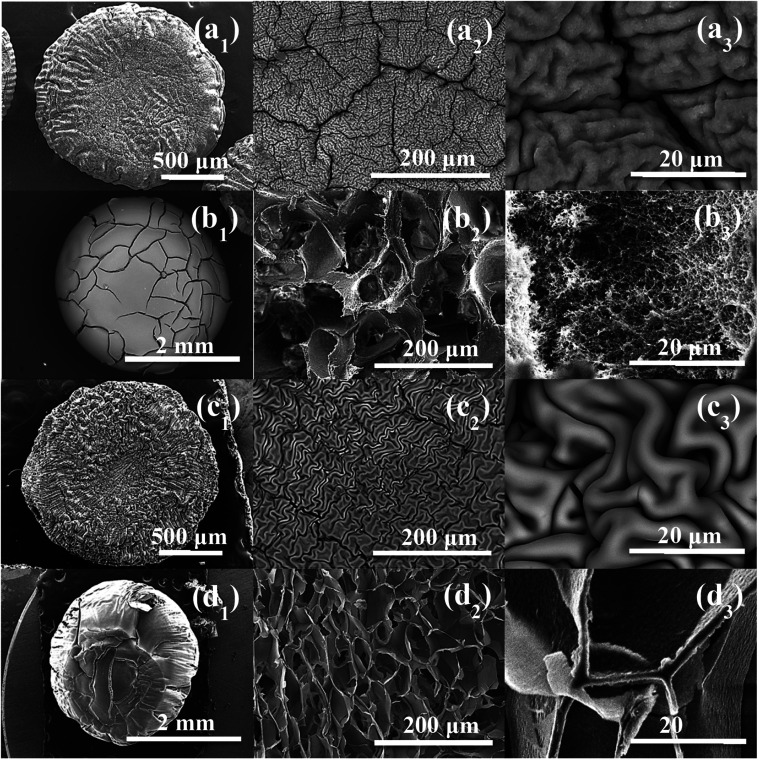
SEM observation of: (a_1_–a_3_) CMC@Ti_X, (b_1_–b_3_) CMC@Ti_C, (c_1_–c_3_) alginate@Ti_X and (d_1_–d_3_) alginate@Ti_C.

The SEM micrographs of the dried hydrogel beads *via* the two different routes were presented in Fig. 3. The xerogels independently of used polymer ((a_1_), (a_2_), (a_3_)) & ((c_1_), (c_2_), (c_3_)) exhibited, at low magnification, lentil-shape morphology (circular but flattened shape). The same applies to (b_1_) and (d_1_).

At high magnification, the cryogel-beads have a surface in the form of folds, which gives it a wrinkled shape. These folds make it possible to obtain a large surface with a limited volume. The SEM images of the cryogel-beads ((b_1_), (b_2_), (b_3_)), when we used CMC as gelling agent, present a foamy texture morphology, with open micrometric pores, which are interconnected through small windows (b_2_). The image (b_3_) confirms the pores connect with the inter-particle spaces. This clearly illustrates that freeze-drying process allow the formation of a highly porous structure. In the case of using alginate, we noted the obtaining of a cellular porous structure (d_2_). The cells are dense, with large ordered windows, and least interconnected walls (d_3_). The interconnections of the pores are more accentuated in the sample, when we used CMC. The drying manner influences thus the morphology of the obtained beads. The freeze-drying process eliminates water by sublimation, without damaging the porous structure, which prevent structural collapse and limit the shrinkage phenomenon. Lyophilization allows the preservation of the spherical shape, color, appearance and morphology.^52,53^

Fig. S1† presents the energy dispersive spectroscopy analysis of hybrid-beads. It should be noted, however, that all samples were composed of carbon, oxygen and titanium atoms. The C element is originated from the gelling agent. The absence of any other impurity proves that chlorine originated from titanium precursor has been removed thorough washing prior to the drying process. These results indicate the high purity of the as-prepared hybrid-materials.

#### 3.1.4 Thermogravimetric analysis of hybrid-beads

**Fig. 4:**
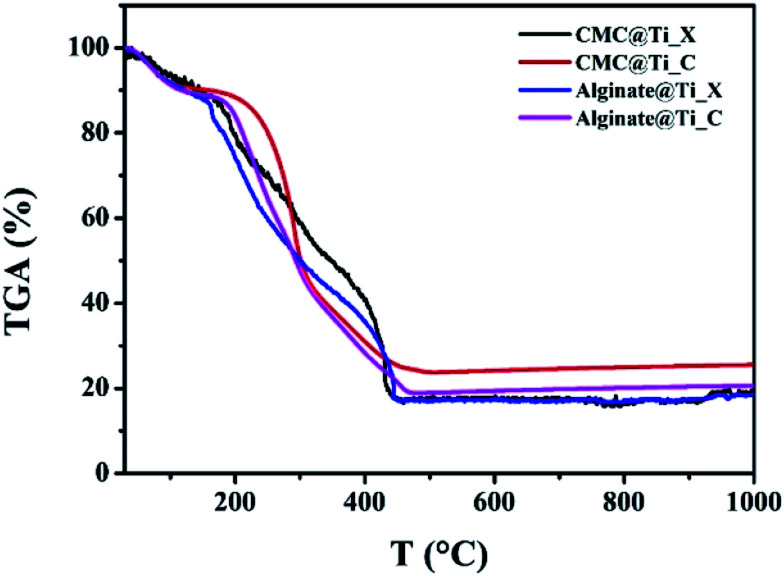
Thermogravimetric analysis of the as-prepared xerogel-, and cryogel-beads.

The TGA of the xerogel and cryogel beads were shown in Fig. 4. These curves indicate the difference in degradation of organic residues inside of both samples (xerogel and cryogel), in order to determine the ideal thermal treatment for titania crystallization. All samples show similar thermal behaviours with three different zones of hybrid-beads decomposition. The first is characterised by its weight loss of 10 to 12%, from ambient temperature to 160°, can be attributed to the evaporation of physisorbed water molecules. The second zone correspond to weight loss between 160 and 350 °C, which was assigned to biopolymers thermal degradation (CMC or alginate). The final zone is between 350 to 500 °C assigned to weight loss of 20 to 24%. This can be explained by the complete removal of residual organic matter. The thermal stability is occurred around 500 °C. Thus, we adopted this temperature for the samples thermal treatment.

### 3.2 Characterization of the elaborated titania

#### 3.2.1 Microstructural characterization

**Fig. 5:**
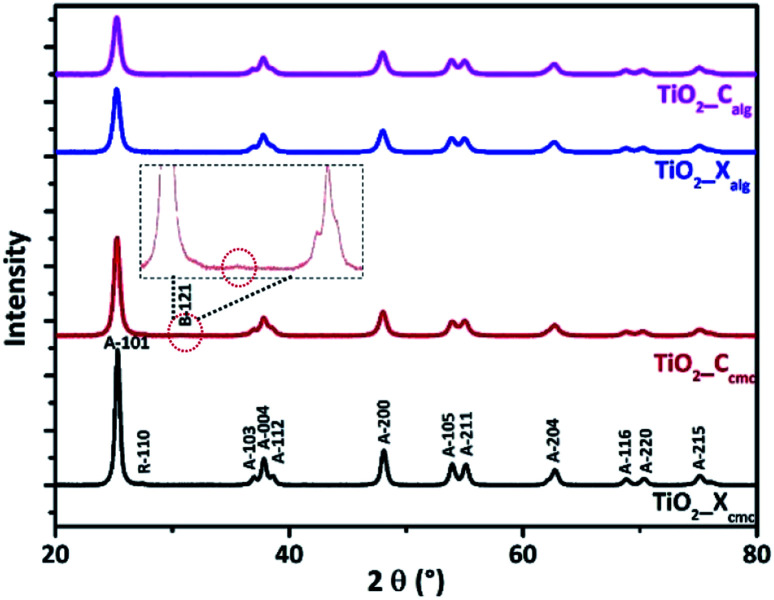
XRD patterns of the as-synthesized TiO_2_.

Fig. 5 shows the XRD diagrams of TiO_2__X_cmc_, TiO_2__C_cmc_, TiO_2__X_alg_ and TiO_2__C_alg_. These analyses revealed that both samples prepared *via* alginate gelation exhibit reflections corresponding to anatase phase (ICDD card no. 96-153-0152, ICDD card no. 96-900-8215). But, in the case of CMC use, we note, moreover, from xerogel the presence of a small peak due to rutile phase (ICDD no. 96-900-7433). The oxide obtained from cryogel-beads show also a new phase concern brookite (ICDD no. 96-900-4141). The presence of intense, and broad peaks indicates the formation of highly nanocrystalline TiO_2_ particles. It is noted that the oxide prepared *via* the CMC's gelation presented higher peaks intensity in comparison to those synthesized using alginate. The effect of biopolymer used as gelling agent and also the drying process is significant on obtained crystalline phases, and their size, which was estimated firstly by the Debye–Scherer formula (eqn (1)):1
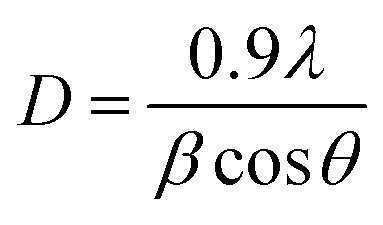
where *D*, in nm, is the crystallite size, *λ* is the X-ray wavelength corresponding to Cu K radiation, *β* in radians is the Full Width at Half Maximum (FWHM) of the XRD peak and *θ* is the diffraction angle.^54^ The results of this calculation are shown in Table 2. The obtained oxides from alginate-beads provides smaller crystallite size in comparison to those obtained from CMC-beads. Both oxides obtained from alginate-beads have approximatively similar crystallite size values independently the drying process (10.43 nm, and 10.35 nm). The size of the crystallites of the oxides obtained from CMC beads is 16.13 nm and 12.68 nm, respectively with conventional drying and freeze-drying. This difference in size between these oxides is possibly related to the difference in the sequence of the marker units (monomer units) of each polymer.

**Table 2:** Crystallite sizes, phase percentage, and surface area as a function of used biopolymers and drying mode

Sample	Crystallite size (nm)	Phase percentage (%)			BET (m^2^ g^−1^)
		Anatase	Rutile	Brookite	
TiO_2__X_cmc_	16.13	97.15	2.85	0	33.04
TiO_2__C_cmc_	12.68	98.18	0	1.82	47
TiO_2__X_alg_	10.43	100	0	0	59.03
TiO_2__C_alg_	10.35	100	0	0	80.04

**Fig. 6:**
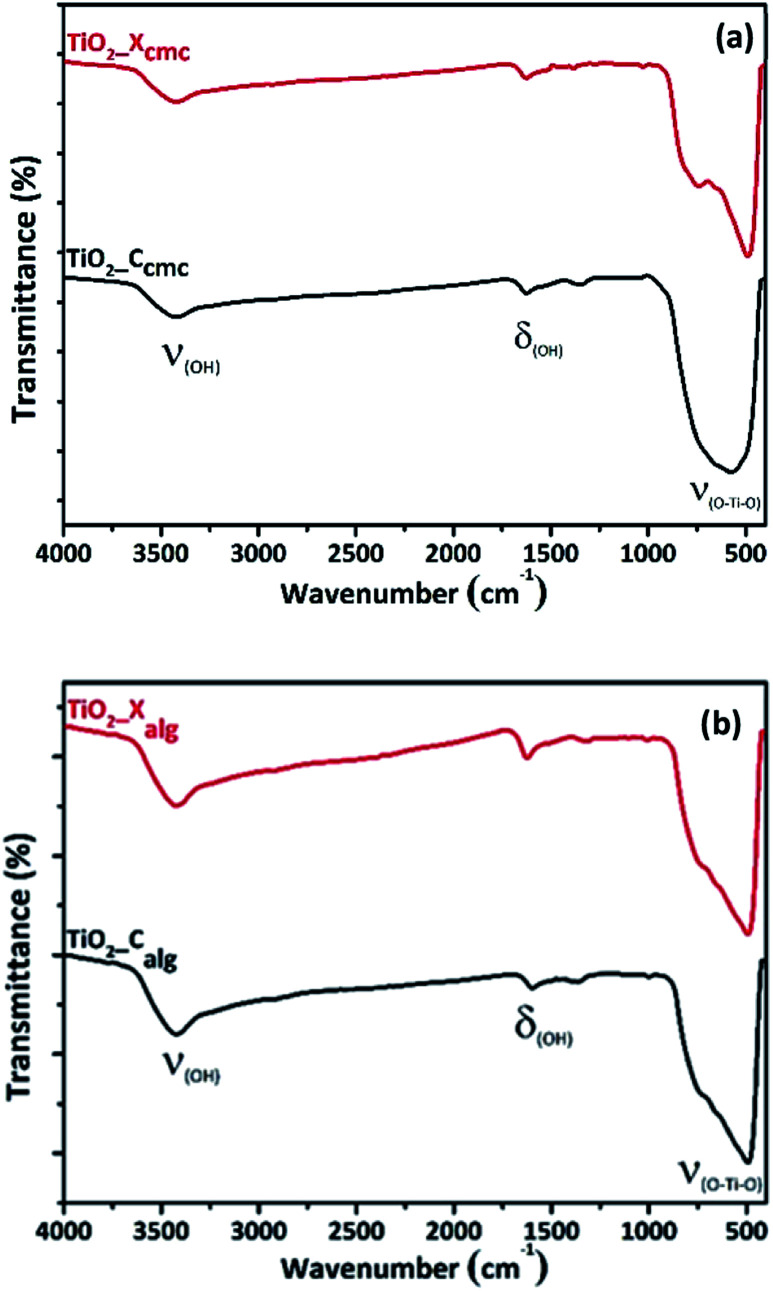
FTIR spectra of oxides elaborated *via* the gelation of: (a) CMC and (b) alginate.

In Fig. 6, the FTIR spectra of TiO_2_ shows three different bands. The first band is observed at 3400 cm^−1^, corresponding to the stretching vibration of the hydroxyl group O–H of the physisorbed water on TiO_2_ surface. The second band is observed around 1630 cm^−1^, corresponding to bending modes of Ti–OH; the last is a prominent and broadest peak at the range of 400–800 cm^−1^ related to Ti–O modes.^55,56^

#### 3.2.2 Morphology and textural analysis

**Fig. 7:**
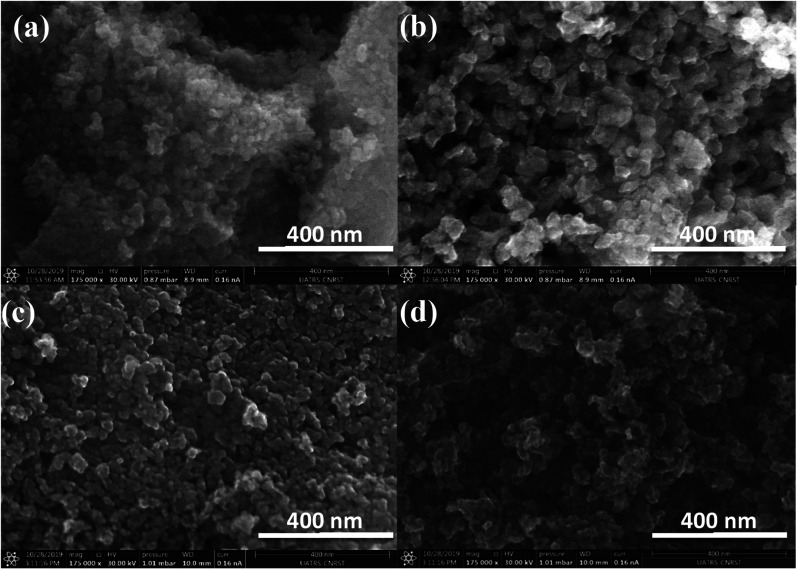
SEM observation of: (a) TiO_2__X_cmc_, (b) TiO_2__C_cmc_, (c) TiO_2__X_alg_ and (d) TiO_2__C_alg_.

SEM images of calcined cryogel-beads show at low magnification the porous structure of the oxides which consists of interconnected pores, especially in the case of TiO_2__C_alg_ (Fig. S3†). So, SEM photos under advanced magnification indicate the surface roughness of all samples, and which takes on remarkable importance in the two samples obtained by freeze-drying independently of biopolymer (Fig. 7). This is illustrated by the anarchic arrangement of the surface particles according to the terrace-step-notch model for a rough surface. The regularity of the structure emerges in these both samples: (b) TiO_2__C_cmc_, and (d) TiO_2__C_alg_. The chemical composition of the oxides were achieved by using the SEM-EDS analysis (Fig. S2†). Those analysis show a strong peak Kα at 0.52 keV corresponding to oxygen and the presence of the characteristic peaks Kα and Kβ of titanium (energy of Kα and Kβ: 4.511 keV and 4.931 keV).^57^ The semi-quantitative results give the stoichiometric proportions of Ti and O elements (38 at% for Ti and 62 at% for O). There was no residual elements from the synthesis chemicals, which confirms the presence of TiO_2_ with high purity.

**Fig. 8:**
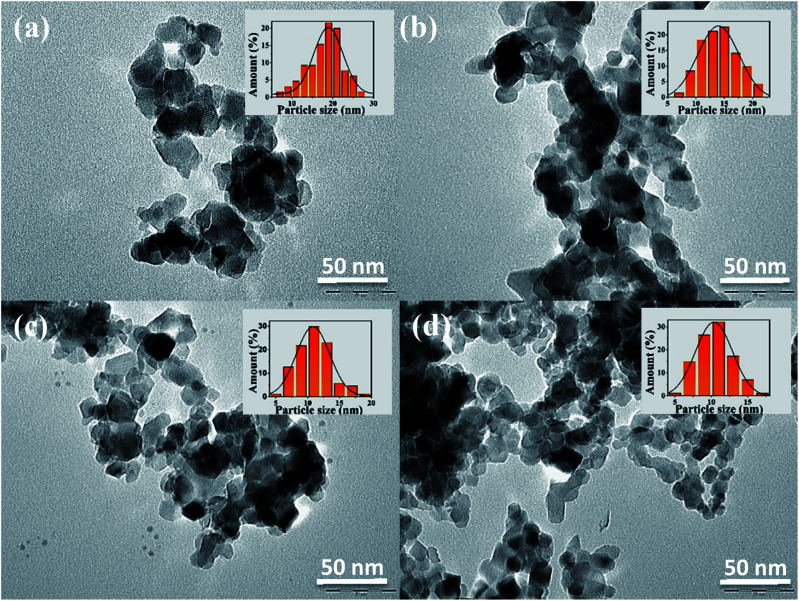
TEM images and particle size distribution histogram of: (a) TiO_2__X_cmc_, (b) TiO_2__C_cmc_, (c) TiO_2__X_alg_ and (d) TiO_2__C_alg_.

In Fig. 8, the TEM images for (a) TiO_2__X_cmc_, (b) TiO_2__C_cmc_, (c) TiO_2__X_alg_ and (d) TiO_2__C_alg_ show that titania nanoparticles are wholesale spherical or egg-shaped, with uneven and heterogeneous dispersion. Nanoparticles are in the form of agglomerated superstructures, which can be explained by the crystallization mechanism and Ostwald ripening inhibition, which allow stabilization of relatively smaller aggregated nanoparticles. The average diameters are of 18.07, 14.18, 11.01 and 10.34 nm for TiO_2__X_cmc_, TiO_2__C_cmc_, TiO_2__X_alg_ and TiO_2__C_alg_, respectively. These results are in good agreement with XRD values. The mean particle size seems to be influenced by the biopolymer nature and the drying process.

The surface areas of titania nanoparticles were presented in Table 2. The surface areas ranged from 33.04, 47, 59.03, to 80.04 m^2^ g^−1^ for respectively TiO_2__X_cmc_, TiO_2__C_cmc_, TiO_2__X_alg_, and TiO_2__C_alg_. The highest surface areas were observed for samples prepared *via* gelation of the alginate, with a predominance of the sample obtained from freeze-drying alginate-beads (TiO_2__X_alg_).

#### 3.2.3 Optical properties

**Fig. 9:**
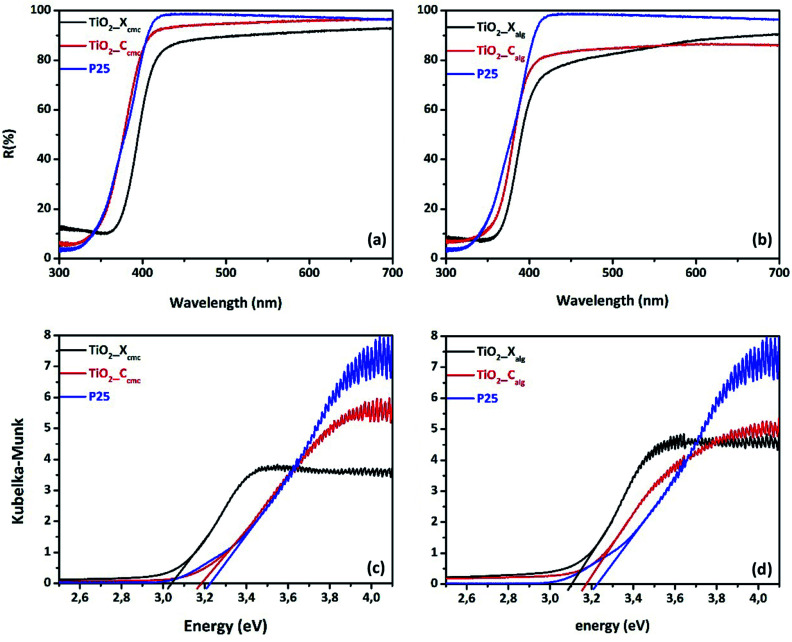
UV-vis diffuse reflectance spectra of TiO_2_ prepared using: (a) CMC, (b) alginate; and the corresponding Kubelka–Munk for oxide elaborated *via* biopolymer gelling: (c) CMC & (d) alginate.

Diffuse reflectance spectroscopy was used to analyze the optical absorption of all oxides. Fig. 9(a) and (c) presents the UV-vis diffuse reflectance spectra of as-synthesized titania and of the commercial P25 as standard sample. All samples showed an intense absorption in the UV region with an absorption edge located around 400 nm. In addition, a shift of the reflectance spectrum towards the visible region was observed for oxides obtained from calcined xerogel-beads, which was more significant for TiO_2__X_cmc_, in comparison to TiO_2__C_cmc_ and the P25. Band-gaps values were calculated using Kubelka–Munk function, according to Tandon–Gupta method.^58^ The bandgap values were estimated from Tauc plot by extrapolating the linear part of the curve, of (*αhν*)^1/2^*versus* energy (*hν*) (Fig. 9(b), (d) and Table 3). All samples presented a less weak bandgap in comparison to pure anatase (*E*_g_ = 3.2 eV) and to P25 (*E*_g_ = 3.22 eV). TiO_2__X_cmc_ has lowest bandgap value. That could be due to the presence of an amount of rutile phase known by its smaller bandgap in comparison to pure anatase phase.^26^

**Table 3:** Bandgap and zeta potential values of the different photocatalysts

Sample	TiO_2__X_cmc_	TiO_2__C_cmc_	TiO_2__X_alg_	TiO_2__C_alg_
Bandgap value (eV)	3.04	3.18	3.1	3.17
Zeta potential (mV)	−29.17	−32.27	−21.53	−37

The zeta potential of all samples in water (pH = 7.4) was also listed in Table 3. The zeta potential values are: −29.17, −32.27, −21.53, and −37 mV for TiO_2__X_cmc_, TiO_2__C_cmc_, TiO_2__X_alg_, and TiO_2__C_alg_, respectively, whereas the zeta potential value for P25 is around −35 mV. The TiO_2__C_alg_ sample reveals the most prominent increase in zeta potential value (−37 mV), which can indicate the excellent stability of TiO_2_ nanoparticles under this pH condition. The negative charges on the surface of all samples are important to degrade Orange G by neglecting the adsorption process due to electrostatic repulsion between negatively charge TiO_2_ and dye molecules (anionic).

## 4. Photocatalytic properties

**Fig. 10:**
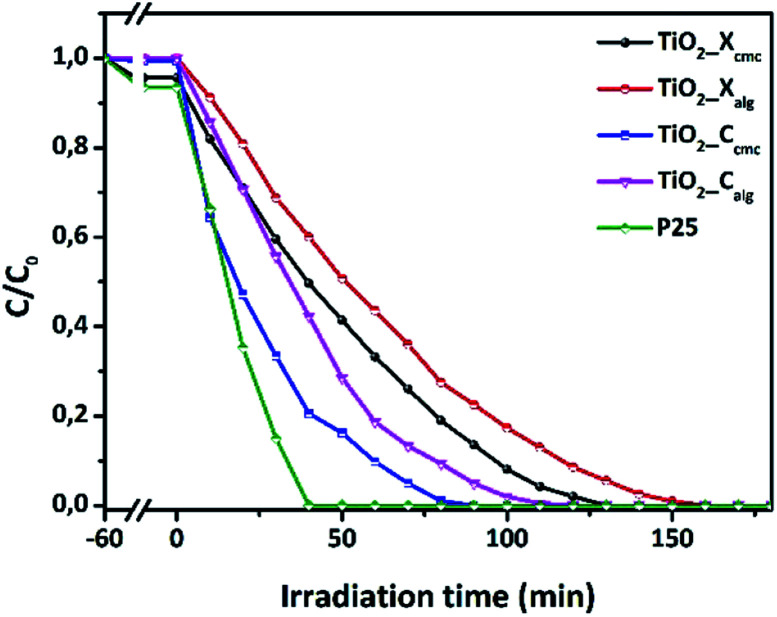
Photocatalytic performances of the four as-prepared oxides in accordance with the synthesis conditions.

The photocatalytic performances of TiO_2_ samples were tested by measuring the degradation of Orange G in aqueous solution at regular time intervals. Fig. 10 shows the initial adsorption and degradation profiles of OG by TiO_2__X_cmc_, TiO_2__C_cmc_, TiO_2__X_alg_, and TiO_2__C_alg_. The adsorption process, conducted under obscure conditions during one hour, show a negligible initial adsorption (<5%). Then, OG solutions were irradiated in the presence of the catalysts. The solutions comprising the azo-dye were almost fully degraded over different time periods such as: 90, 120, 130 and 160 min, corresponding to TiO_2__C_cmc_, TiO_2__C_alg_, TiO_2__X_cmc_ and TiO_2__C_alg_, respectively. Thus, it appears clearly that the sample obtained from CMC-beads freeze-drying has the best photocatalytic performance. However, it should be noted that this sample contains a mixed anatase and brookite crystallographic phases, which is in agreement with the bibliography data.^28^ This can be explained by the increase in facets since photocatalysis is, amongst others, a surface phenomenon.

The OG photocatalytic degradation kinetics were studied using the pseudo-first-order kinetic model, which is expressed as follows:2
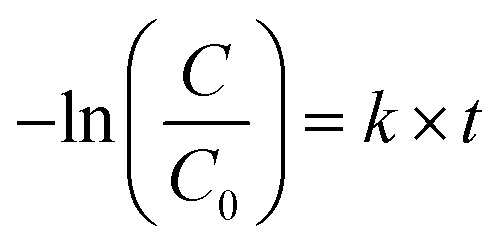
where, *C*_0_ and *C* are the initial and the exhaustion OG concentration, respectively, *k* is the reaction rate constant and *t* is the reaction time.

**Fig. 11:**
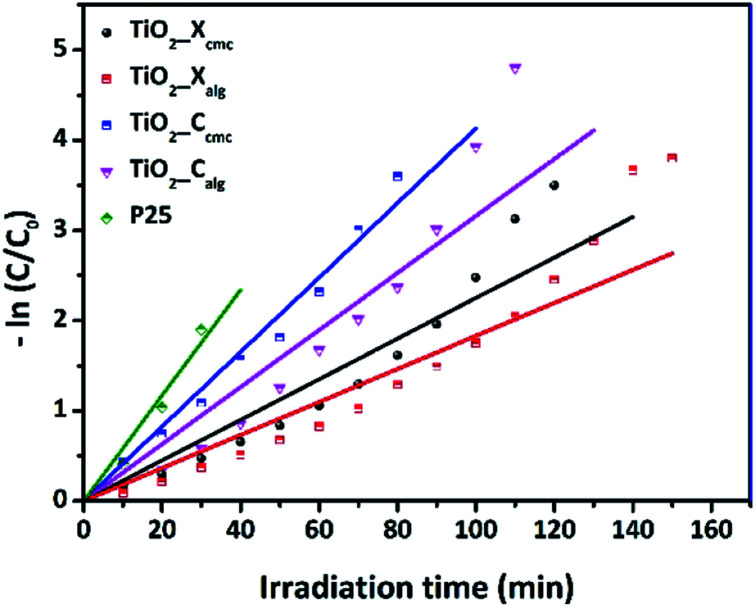
Kinetics of OG degradation reaction photo-catalyzed by the as-prepared samples and P25.

Fig. 11 presents the plot of ln(*C*_0_/*C*) *versus t*. The pseudo-first-order rate constants (*k*) were determined by the slope of the kinetic curves and are summarized in Table 4. The correlation coefficient *R*^2^ ranges between 0.915 and 0.98. Thus, the first-order model fits quite well the experimental photocatalytic evaluation data. The rate constant were 4.13 × 10^−2^, 3.16 × 10^−2^, 2.25 × 10^−2^, and 1.83 × 10^−2^ min^−1^ for TiO_2__C_cmc_, TiO_2__C_alg_, TiO_2__X_cmc_, and TiO_2__X_alg_, respectively. This revealed that TiO_2__C_cmc_ has the highest degradation rate and got closer to that of the commercial product P25.

**Table 4:** Kinetic constants of OG degradation reaction photo-catalyzed by nanostructured TiO_2_

Sample	*k* (×10^−2^ min^−1^)	Linear regression (*R*^2^)
TiO_2__X_cmc_	2.25	0.915
TiO_2__C_cmc_	4.13	0.980
TiO_2__X_alg_	1.83	0.935
TiO_2__C_alg_	3.16	0.922
P25	5.85	0.967

## 5. Discussions

In the current investigation, we evaluated the effect of the gelling agent and the drying way in order to control growth of nanostructured titania. Both polymers used in this study (alginate and CMC) have the capacity to form hydrogels in the presence of cations. It is a gelation by ionotropic effect, involving interactions between charges of the Ti^4+^ cations and carboxylate functions, and the oxygen atoms of the hydroxyl functions wear by monomers of biopolymer chains, in order to form hydrogel's three-dimensional network. The egg-box model is the most commonly used representation of the interactions between alginate or CMC chains and cations (Fig. S6 and S7†). The affinity of the chelating carboxylic groups and hydroxyl functions with Ti^4+^ cations certainly depends on their spatial distribution in each biopolymer. The difference observed between the beads produced by alginate gelation or by CMC is due to the difference in the interaction of Ti^4+^ cations with each biopolymer, which implies variant of the conformational transition of chains in each polysaccharide. The freeze-drying process allowed the preservation of the tri-dimensional hydrogel structure. This technique consists in removing water from the hydrogel by the combined action: cold and vacuum. Freeze-drying generally involves three stages: freezing, sublimation and drying. The water heated in the solid state and at very low pressure sublimes and thus goes directly from the solid-state to the gaseous state. This technique maintains the volume, appearance and distribution of crosslinked biopolymer fibrils. On the other hand, conventional drying at room temperature under air damages the hydrogel structure *via* the capillarity's collapse due to the surface tension of the liquid–gas–solid junction in the hydrogel which contains about 80% water. The difference between xerogel-, and cryogel-beads affects consequently the properties of the corresponding oxides. Microstructural analysis of oxides by FTIR reveals the presence of the fingerprints of this oxide. From a crystallographic point of view, the use of alginate as gelling agent gives rise a pure anatase phase titania. But, when we used CMC as structuring agent, we obtained a mixed phases: anatase–rutile and anatase–brookite for TiO_2__X_cmc_ and TiO_2__C_cmc_, respectively. This can be explained by the difference in gelling mechanism of each biopolymer, especially the conformational transition of their crosslinked chains, and then their association to form the outer-shell of the hydrogel. It is obvious from this analysis that the drying method has also an influence on the second crystallographic phase nature (minor phase). The valence band consists of the p states of oxygen, and the conduction band of the d states of titanium. The titanium dioxide in its anatase form has a bandgap of 3.26 eV (380 nm), in its rutile form the bandgap is 3.05 eV (407 nm) and finally its brookite form with a bandgap of 3.14 eV (395 nm).^26^ The bandgap values of the oxides obtained by the method we developed here, are: 3.04, 3.18, 3.1, and 3.17 eV for TiO_2__X_cmc_, TiO_2__C_cmc_, TiO_2__X_alg_, and TiO_2__C_alg_, respectively. All as-prepared samples present bandgap values lower than the theoretical one. The values vary according to the gelling agent and the drying route. It should be noted that the oxides presenting a mixed phases (anatase and rutile) can have lower bandgap value.^24,59–61^ The oxides TiO_2__C_cmc_ and TiO_2__C_alg_ present the high energy values of 3.18 and 3.17 eV, respectively. TiO_2__X_cmc_ sample has the lowest bandgap of 3.04 eV, which is lower than the commercial one P25. This sample contains apart anatase phase a small amount of rutile phase. It is a very interesting result for the visible-light activation.

Through its various structural, electronic and optical properties presented above and due to the ease of their synthesis, their chemical stability, and their efficiency, TiO_2_ samples were tested as photocatalysts for oxidizing OG in order to validate the core concept. The photocatalytic activity of produced samples is affected by its physicochemical properties in particular the crystal structure. The anatase phase is often considered to be the most photoactive. However, this conclusion is still subject to debate. The anatase has an indirect bandgap, which makes it difficult to transition from the photoexcited electron from the conduction band to the valence band. While the bandgaps for rutile and brookite are direct bands. The lifetime of electrons and holes is longer in the anatase phase.^62^ The effective mass of the electrons, present in the anatase phase, is lighter than for the two other crystalline forms, which induces a rapid migration of the charge carriers to the surface, thus reducing their recombination velocity.^62^ Another element explaining the difference in photoactivity is the level of Fermi energy, which is slightly higher in the anatase. This induces a decrease of the affinity with oxygen and an increase of the number of hydroxyl groups on the surface contributing to a higher photocatalytic activity.^63^ However, the mixture of the two anatase and rutile phases in the case P25 (anatase/rutile: 80/20) is often found in the literature as the most effective structure compared to the pure anatase and rutile phases.^64^ The presence of a junction between anatase/rutile and anatase/brookite is the reason for the important effectiveness of mixed-phases.^65^ This interface would allow the transfer of electrons from the conduction band of the anatase to the conduction band of the rutile. In addition, the brookite has an orthorhombic structure (unit cell) containing eight TiO_2_ patterns in which the vertices and edges of the octahedron were shared. The average length of the Ti–O bonds is 1.93, 1.96, and 1.87 Å, in the anatase, rutile, and brookite, respectively.^66^ In our case, the best performing sample is that containing the anatase and the brookite phases. These preliminary results show that the photocatalytic process is directly correlated with the crystallographic structure of the oxides. We then noticed that under irradiation, the sample with the largest specific surface (TiO_2_-C_alg_) has a certain efficiency compared to the other samples. This point therefore remains debated.

## 6. Conclusion and perspectives

Nanocrystalline titania was successfully elaborated *via* the biopolymers gelation. The synthesis conditions (biopolymer nature, drying mode) demonstrated to have a great impact on structural, morphological, optical and electronic properties of the as-elaborated oxides. Phase-pure anatase was obtained by using alginate whereas mixed TiO_2_ polymorphs, with a relatively higher particle size, were obtained *via* CMC. Lyophilized hybrid-beads have the highest bandgap values, porosity, surface areas and photoactivities. However, the conventional drying route induces a promising visible photoactive material, TiO_2__X_cmc_, with the lowest bandgap value of 3.04 eV. The performed photocatalytic studies demonstrated that the photo-oxidation of OG was improved by mixed-phase TiO_2_. TiO_2__C_cmc_ (anatase–brookite) was the best performing sample toward OG degradation. Different perspectives open up as a result of this work: (i) it would be interesting to optimize the synthesis parameters in order to well control growth of this oxide in mixed phases so as to take better advantage of the synergistic effect mentioned in the literature, and proceed towards doping TiO_2_ by other elements. An interesting option is to test other types of biopolymers and also other drying technologies; and (ii) from the photocatalysis point of view, the photocatalytic degradation of dyes of various types (anionic, cationic, and nonionic) should be achieved by studying all the parameters of this process.

## Conflicts of interest

There are no conflicts to declare.

## Supplementary Material

RA-010-D0RA03312J-s001
