# Supplementary material for: Controlling the growth of nanosized titania via polymer gelation for photocatalytic applications
Source: RSC Adv. 2020 May 21;10(33):19443–53. doi: 10.1039/d0ra03312j (PMC9054054; doi:10.1039/d0ra03312j)
Supplement: RA-010-D0RA03312J-s001 [file RA-010-D0RA03312J-s001.pdf]

Electronic Supplementary Material (ESI) for RSC Advances.
This journal is © The Royal Society of Chemistry 2020

ESI (electronic supplementary information)

Title:

Controlling growth of nanosized titania *via* polymers gelation for photocatalytic application

Authors:

Yousra El Jemli,[a,b] Mohammed Mansori,[a] Oscar Gonzalez Diaz,[b] Abdellatif Barakat,[c,d] Abderrahim Solhy,[d] and Karima Abdelouahdi[a*]

Affiliation:

[a] IMED-Lab, FST, Cadi Ayyad University, Marrakesh, Morocco.

[b] Grupo de Fotocatálisis y Espectroscopía para Aplicaciones Medioambientales (Grupo FEAM), Unidad Asociada al CSIC, Dpto. Química, Instituto de Estudios Ambientales y Recursos Naturales i-UNAT, Universidad de Las Palmas de Gran Canaria, Campus Universitario de Tafira, 35017 Las Palmas, Spain.

[c] IATE, Montpellier University, INRAE, Agro Institut, 34060 Montpellier France.

[d] Mohammed VI Polytechnic University, Ben Guerir, Morocco.

Corresponding Author:

E-mail: abdelouahdi@gmail.com

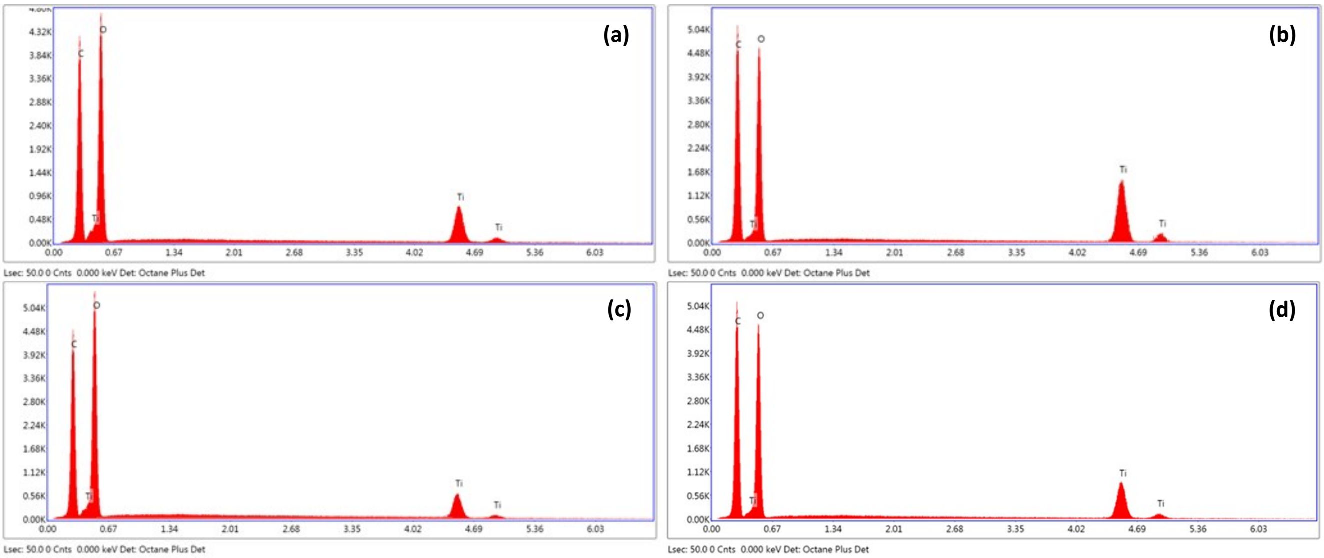

Fig. S1. EDS analysis of: (a) CMC@Ti_X, (b) CMC@Ti_C, (c) Alg@Ti_X and (d) Alg@Ti_C

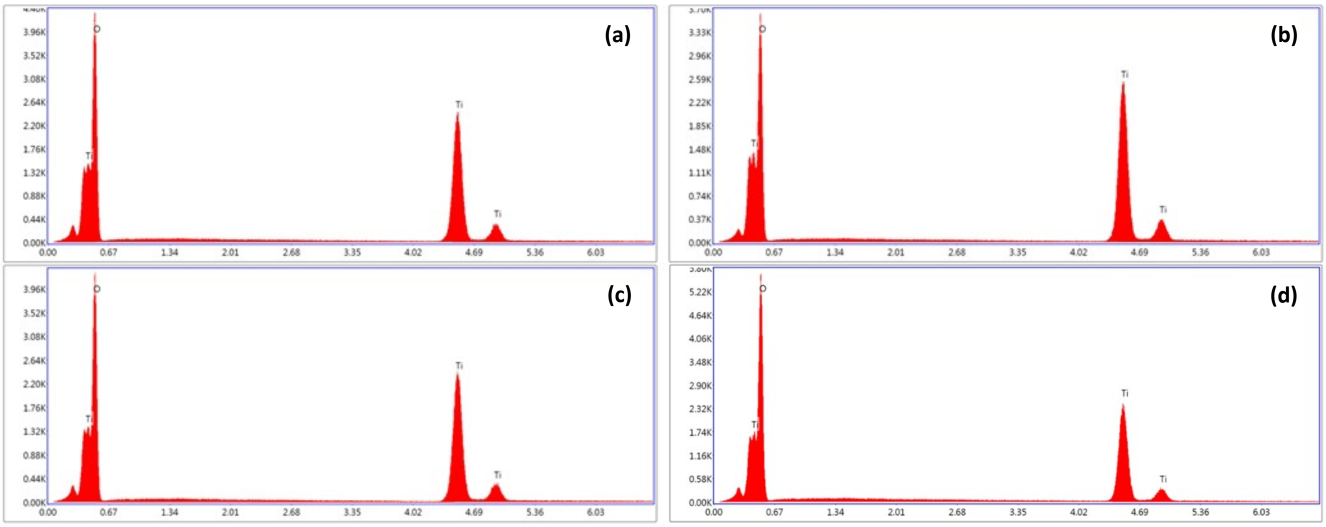

Fig. S2. EDS analysis of: (a) TiO_2_X_{cmc}, (b) TiO_2_C_{cmc}, (c) TiO_2_X_{alg} and (d) TiO_2_C_{alg}

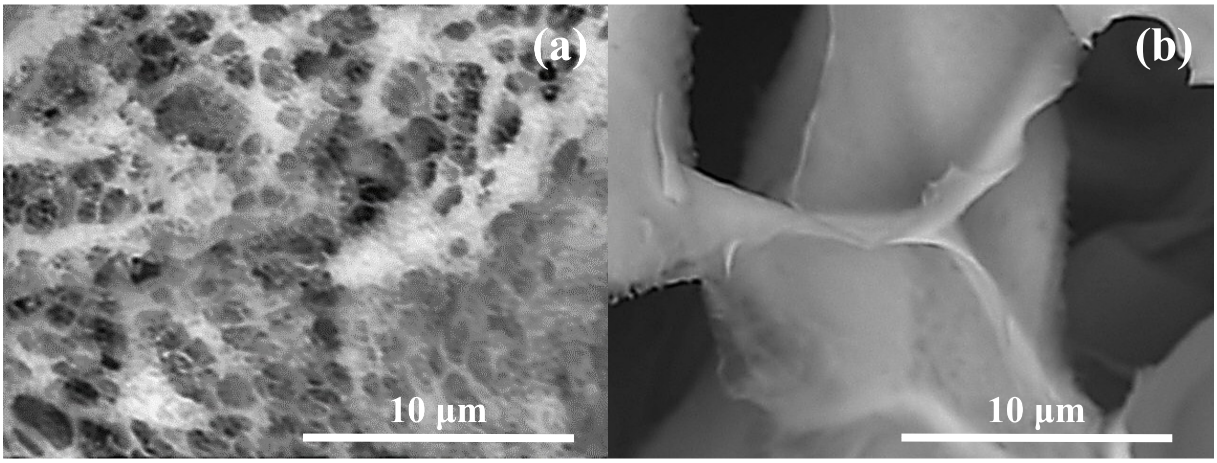

Fig. S3. Low magnification SEM images of: (a) TiO_2_C_{cmc}, and (b) TiO_2_C_{alg}

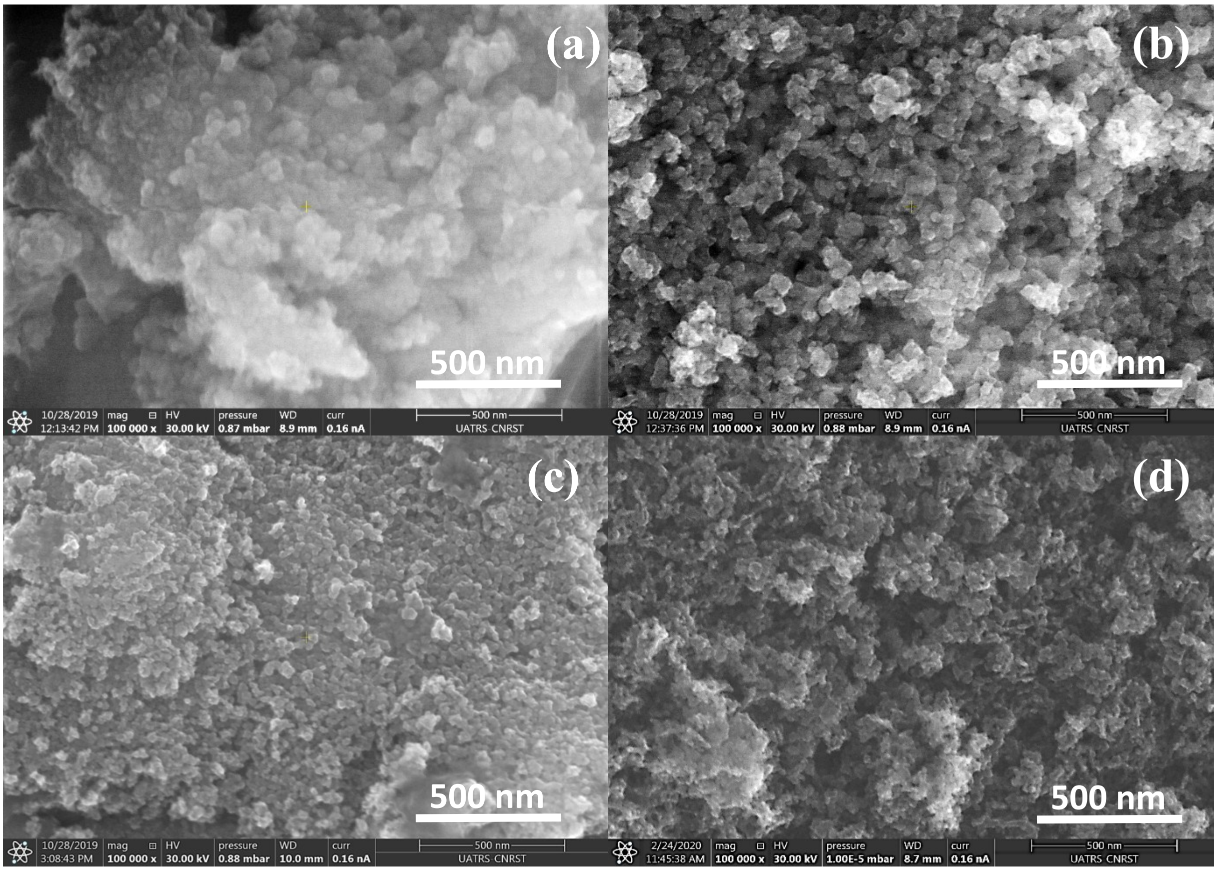

Fig. S4. SEM images of: (a) TiO_2_X_{cmc}, (b) TiO_2_C_{cmc}, (c) TiO_2_X_{alg} and (d) TiO_2_C_{alg}

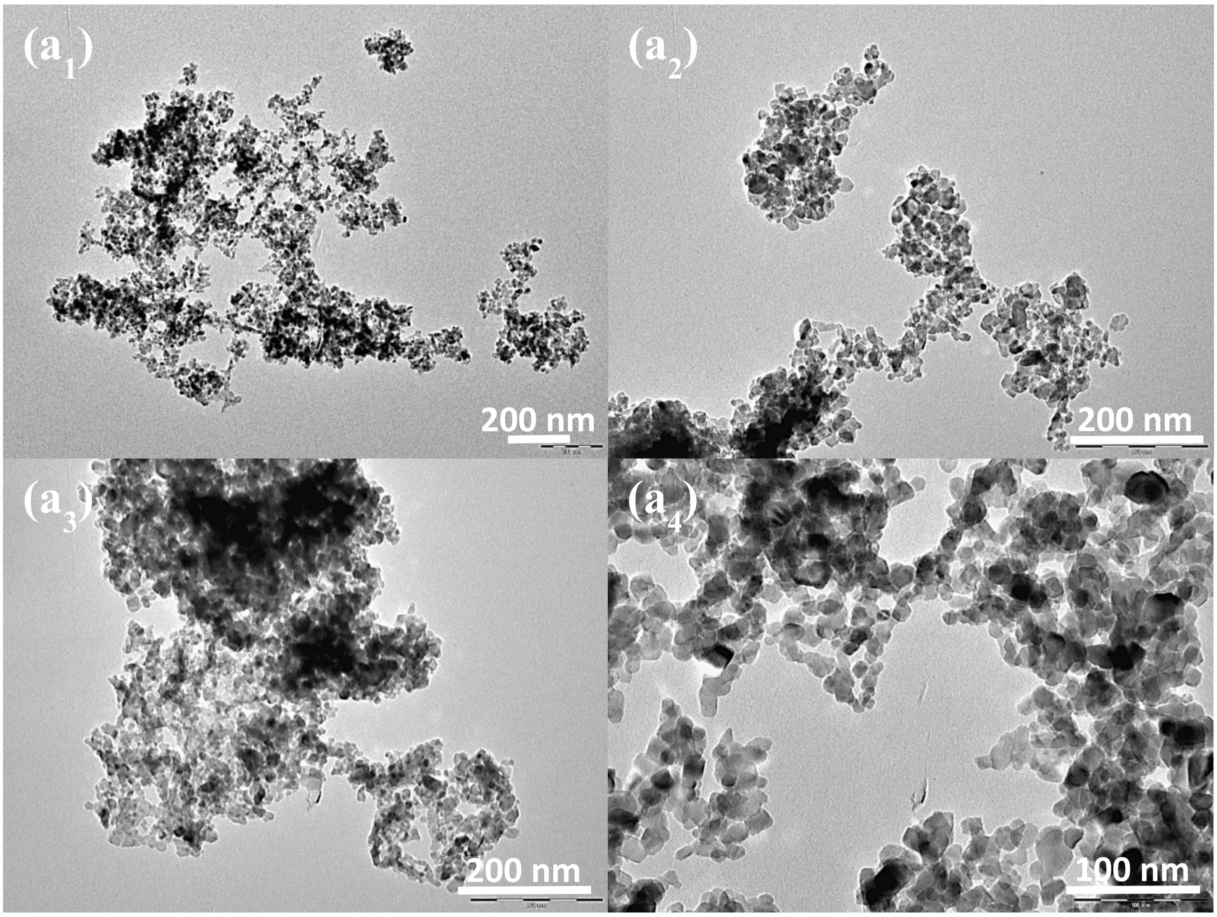

Fig. S5. TEM images of TiO_2_C_{alg} at different magnifications

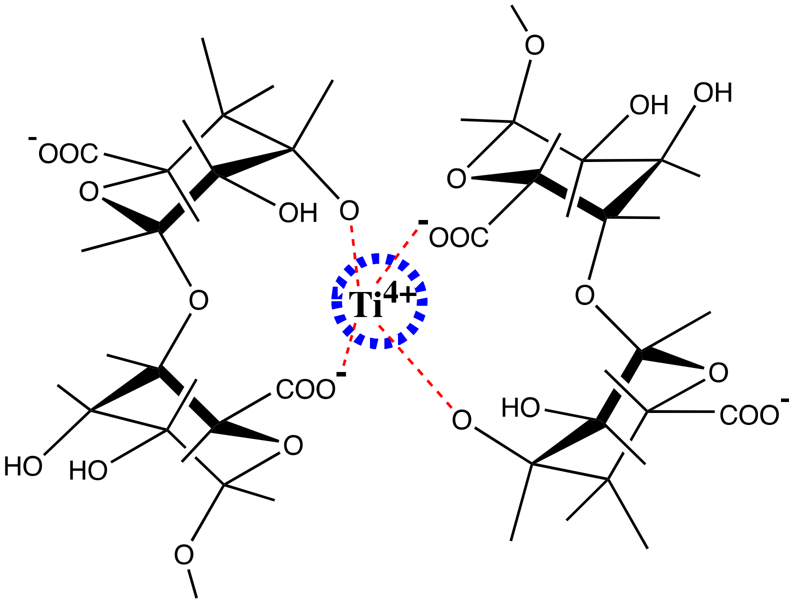

Fig. S6. Ti^{4+} cation chelation by alginate gel

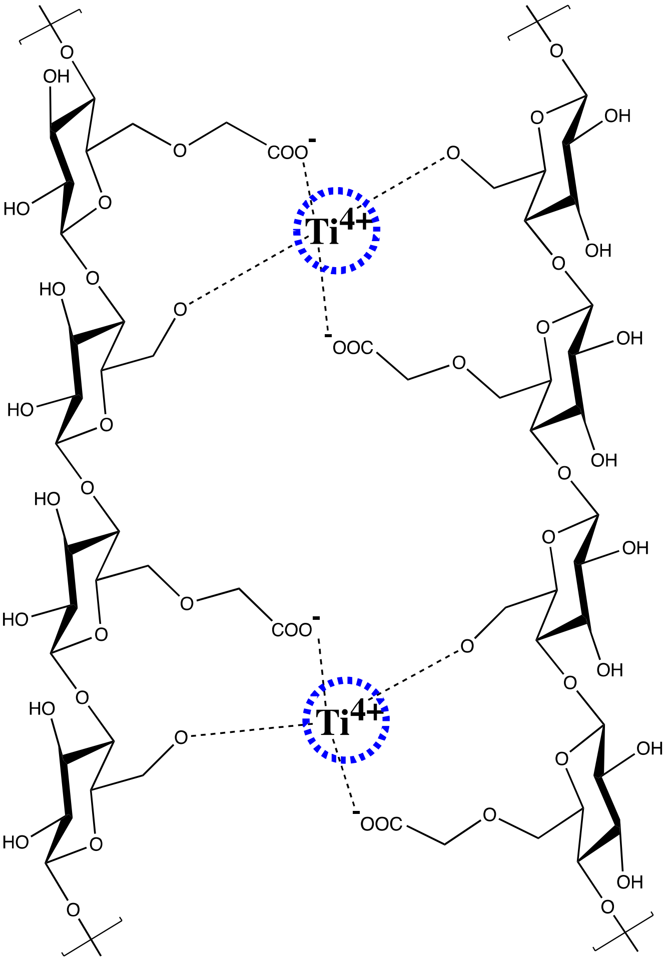

Fig. S7. Ti^{4+} cation chelation by CMC gel
